# Radiation-induced changes in the glycome of endothelial cells with functional consequences

**DOI:** 10.1038/s41598-017-05563-y

**Published:** 2017-07-13

**Authors:** Cyprien Jaillet, Willy Morelle, Marie-Christine Slomianny, Vincent Paget, Georges Tarlet, Valérie Buard, Sonia Selbonne, Fanny Caffin, Emilie Rannou, Pierre Martinez, Agnès François, François Foulquier, Fabrice Allain, Fabien Milliat, Olivier Guipaud

**Affiliations:** 1 Institute for Radiological Protection and Nuclear Safety (IRSN), PRP-HOM, SRBE, L3R, 92260 Fontenay-aux-Roses, France; 2 University of Lille, CNRS, UMR 8576 - UGSF - Unité de Glycobiologie Structurale et Fonctionnelle, 59000 Lille, France; 3 Department of Molecular, Cell and Developmental Biology, UCLA, CA 90095-7239 Los Angeles, USA; 4 GSK - GlaxoSmithKline, 1300 Wavre, Belgium

## Abstract

As it is altered by ionizing radiation, the vascular network is considered as a prime target in limiting normal tissue damage and improving tumor control in radiation therapy. Irradiation activates endothelial cells which then participate in the recruitment of circulating cells, especially by overexpressing cell adhesion molecules, but also by other as yet unknown mechanisms. Since protein glycosylation is an important determinant of cell adhesion, we hypothesized that radiation could alter the glycosylation pattern of endothelial cells and thereby impact adhesion of circulating cells. Herein, we show that ionizing radiation increases high mannose-type N-glycans and decreases glycosaminoglycans. These changes stimulate interactions measured under flow conditions between irradiated endothelial cells and monocytes. Targeted transcriptomic approaches *in vitro* in endothelial cells and *in vivo* in a radiation enteropathy mouse model confirm that genes involved in N- and O-glycosylation are modulated by radiation, and *in silico* analyses give insight into the mechanism by which radiation modifies glycosylation. The endothelium glycome may therefore be considered as a key therapeutic target for modulating the chronic inflammatory response observed in healthy tissues or for participating in tumor control by radiation therapy.

## Introduction

Radiation therapy is used to treat a variety of cancers, as well as benign disorders, in more than half of patients with tumors^1^. Despite tremendous advances in radiation dose delivery techniques, the therapeutic index of radiation therapy is still limited by normal tissue injury in organs at risk and by the radiation resistance of some tumors^2^. Therefore, efforts to develop new approaches to optimize the response of normal tissue and tumors remain essential for improving the outcomes of radiation therapy, by increasing the likelihood of cancer cure or by decreasing normal tissue toxicity, or both^3, 4^.

The vasculature plays a crucial role in tumor progression and in tumor sensitivity or resistance and is considered as a target in attempts to destroy tumors^5^. On the other hand, the vasculature is required for normal tissue homeostasis and also orchestrates wound healing in the case of injury^6^. In the vascular network, the endothelium is considered as a key cell compartment for the response to ionizing radiation of healthy tissue and tumors, and as a promising target to improve the differential effect of radiation therapy in the future^4^. Following stress such as radiation exposure, the global endothelial cell response covers a wide range of molecular events. Changes occur at the transcriptional, translational and post-translational levels and impact cell phenotype, but also the microenvironment, by production and secretion of soluble factors such as reactive oxygen species, chemokines, cytokines and growth factors^7^. These radiation-induced dynamic modifications of molecular networks may control the endothelial cell phenotype and govern recruitment of immune cells.

Ionizing radiation induces an inflammatory response in organs^8^ and tumors^9^ characterized by immune cell infiltration. Vascular endothelium plays an integrative role in the tissue response following stress, and controls the initiation and resolution of inflammatory responses through the regulation of chemotaxis and activation of leukocytes in the periphery^10^. The development of this inflammatory response is regulated by a complex process that involves leukocyte-endothelium interactions composed of activation, rolling, adhesion and transmigration in the surrounding tissue^10^. Engaging the immune system for optimal anti-cancer therapy is an attractive contemporary concept^11^. Promising current strategies generate an effective immune response to destroy the tumor in combination with radiation therapy^12^. In this context, control of the adaptive immune response by the tumor endothelium is a crucial process. By specific expression of a panel of adhesion molecules, as cell adhesion molecules, integrins and selectins, vascular endothelial cells act as a barrier regulating immune cell trafficking on the surface vessel and subsequent extravasation or transmigration. Moreover, secretion of chemokines, cytokines and growth factors contributes to endothelial permeability^9^. The endothelium is thus able to activate a global molecular program in physiological conditions or in response to stress and serves as a key checkpoint to control the immune response. For all these reasons, the vascular endothelium can be considered as a principal checkpoint for radiation-induced inflammatory and immunity processes following radiation exposure in both normal and tumor tissues.

Protein glycosylation of both endothelial and blood circulating cells, as well as endothelial glycocalyx, plays fundamental roles in immune cell trafficking during acute and chronic inflammation^13–15^. Successful therapies for modulating leukocyte-endothelial interactions in human inflammatory diseases may target adhesion molecule glycoforms. Surprisingly, the existence and role of such protein modifications of endothelium in response to ionizing radiation have never been investigated. We and others previously showed that glycosylation of serum proteins is deeply modified in response to localized dorsal radiation injury^16^ and to radiation therapy^17^. These modifications could occur through modifications in expression of genes involved in glycosylation in the liver^16^. Such modifications might have an inhibitory effect on the selectin-mediated entry of leukocytes into inflamed areas through the endothelium^18–20^ and might represent a feedback response of the hepatic acute phase reaction in an attempt to reduce the cellular inflammatory reaction. However, to our knowledge, this hypothesis has so far neither been tested nor proved.

Here, we show using fluorescence microscopy, flow cytometry, mass spectrometry and targeted transcriptomics that ionizing radiation deeply modified the glycome of primary human endothelial cells. These modifications were in part responsible for the radiation-induced increase of monocyte-endothelium interactions under flow, as shown by videomicroscopy experiments. Collectively, these results suggest that regulation of the endothelium glycome is an important process for the adhesion of circulating cells in either healthy tissues or tumors exposed to ionizing radiation.

## Results

### Ionizing radiation increases the level of high mannose N-glycans

**Figure 1:**
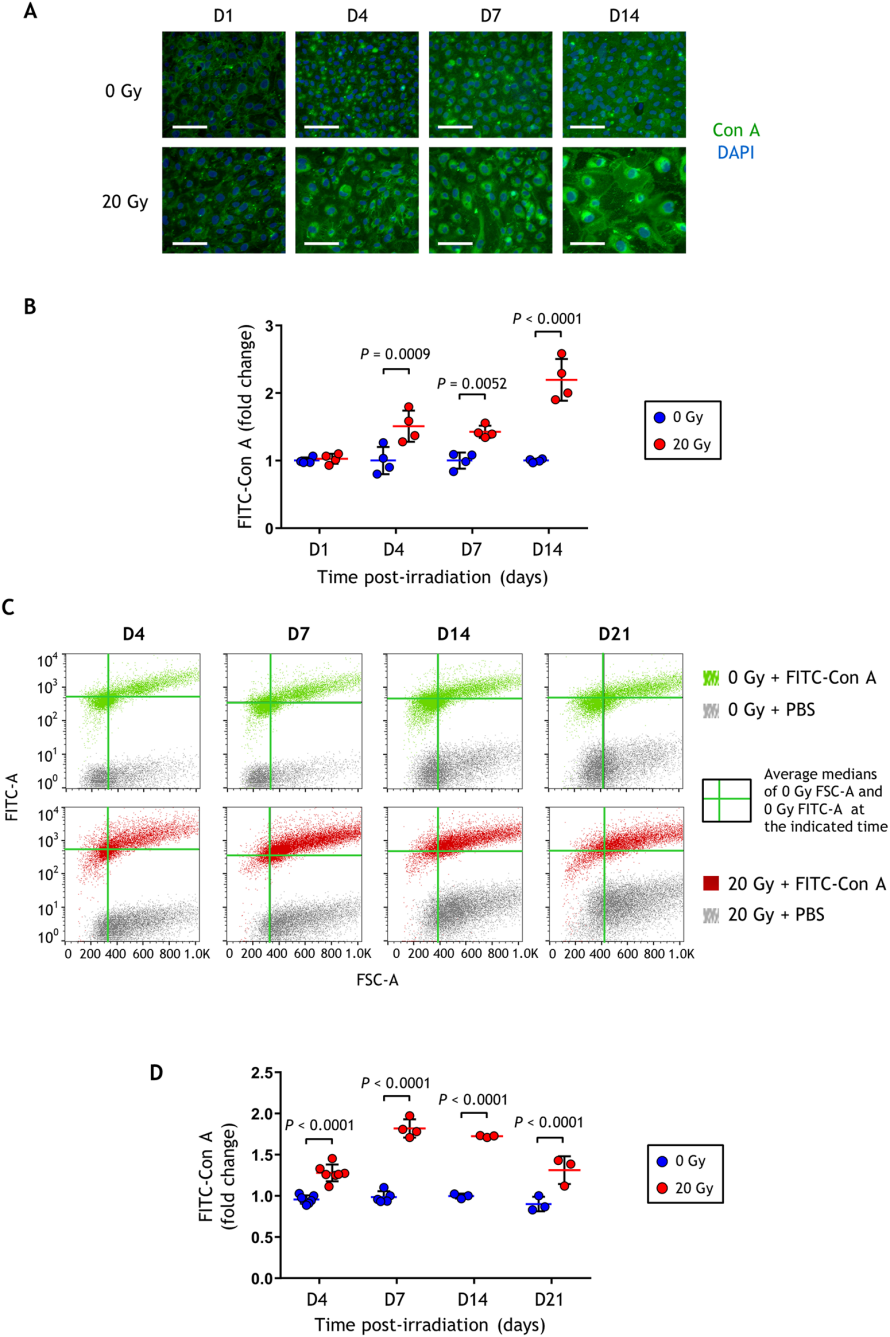
Quantification of Con A staining of HUVECs after irradiation. (**A**) Representative fluorescence microscopy images of HUVECs labeled with FITC-Con A. Scale bars: 100 µm. (**B**) Fold changes of FITC-Con A fluorescence emission densities determined by fluorescence microscopy (mean +/− SD). The experiment was replicated twice (two different cultures) with n = 4 repeated measures within each replication for each time point. Data represent one of the two experiments. (**C**) Biparametric flow cytometry analysis (FSC/FITC-Con A) of HUVECs (one representative experiment). The green solid lines indicate the average medians of the FSC-A (vertical bar) and the FITC-A (horizontal bar) of the control samples at the indicated time post-irradiation. (**D**) Fold change of FITC-Con A fluorescence intensity medians determined by flow cytometry (mean +/− SD). The experiment was replicated twice (two different cultures) at day 4 with n = 4 and n = 3 repeated measures within each replication, and once at days 7, 14 and 21 with n = 4 (D7) or n = 3 (D14 and D21) repeated measures. Data were analyzed by two-way ANOVA.

The response of endothelial cells to radiation exposure leads to a long-term radiation-induced dysfunction phenotype^21^, resulting in greater adhesion of circulating cells^22, 23^. To address whether these radiation-induced changes include changes in glycosylation, HUVECs at confluency were irradiated at 20 Gy and studied from day 1 to day 21 post-exposure. We first used fluorescein-conjugated concanavalin A (FITC-Con A) lectin to quantify high mannose glycan structures in irradiated HUVECs, compared with non-irradiated HUVECs. This lectin tightly binds oligomannose- and hybrid-type N-glycans, but also interacts weakly with biantennary complex-type N-glycans^24^ (Supplementary Fig. S1A). As shown by fluorescence microscopy, the intensity of FITC-Con A on the outer cell membrane of HUVECs increased from day 4 after irradiation and as function of time (Fig. 1A). We tested the specificity of FITC-Con A staining by using the competitive inhibitor α-methylmannose, which clearly caused a complete disappearance of the fluorescence signal (Supplementary Fig. S2A). Since the FITC-Con A staining increased after irradiation, we checked that this was not due to an increase of plasma membrane permeabilization induced by radiation. Cells were stained with the red fluorescent phalloidin conjugate after applying the standard FITC-Con A staining protocol (*i.e*. without permeabilization of plasma membranes). Using fluorescence microscopy, no labeling of F-actin was observed after irradiation unless the cells were permeabilized with Triton X-100 before staining (Supplementary Fig. S2B). This result shows that radiation alone did not permeabilize plasma membranes and suggests that Con A bound specifically to plasma cell membranes and not to intracellular structures.

As HUVECs were increasingly larger in size and less numerous after irradiation, the variation of fluorescence levels was evaluated per unit area of plasma cell membrane rather than fluorescence levels per cell. We show that HUVECs displayed an increase in FITC-Con A intensity per µm² of plasma cell membrane at days 4, 7 and 14 post-irradiation, reaching a maximum fold change of about 2 at day 14 (Fig. 1B). Consistent with these results, flow cytometry analyses of living HUVECs showed an increase of FITC-Con A staining after irradiation, as attested by cell size (FSC)/FITC-Con A bi-parametric analyses (Fig. 1C and Supplementary Fig. S3 for the gating strategy for analysis). Figure 1D displays fold changes of fluorescence median distributions, indicating a clear increase in FITC-Con A staining following radiation exposure. Interestingly, flow cytometry showed a persistent effect 3 weeks after irradiation. Altogether, fluorescence microscopy and flow cytometry data with FITC-Con A show that irradiated HUVECs expressed more high mannose N-glycans on their outer cell membranes than non-irradiated cells, especially at the late time point.

### Irradiation increases the proportion of oligomannose N-glycans at the expense of complex-type structures

**Figure 2:**
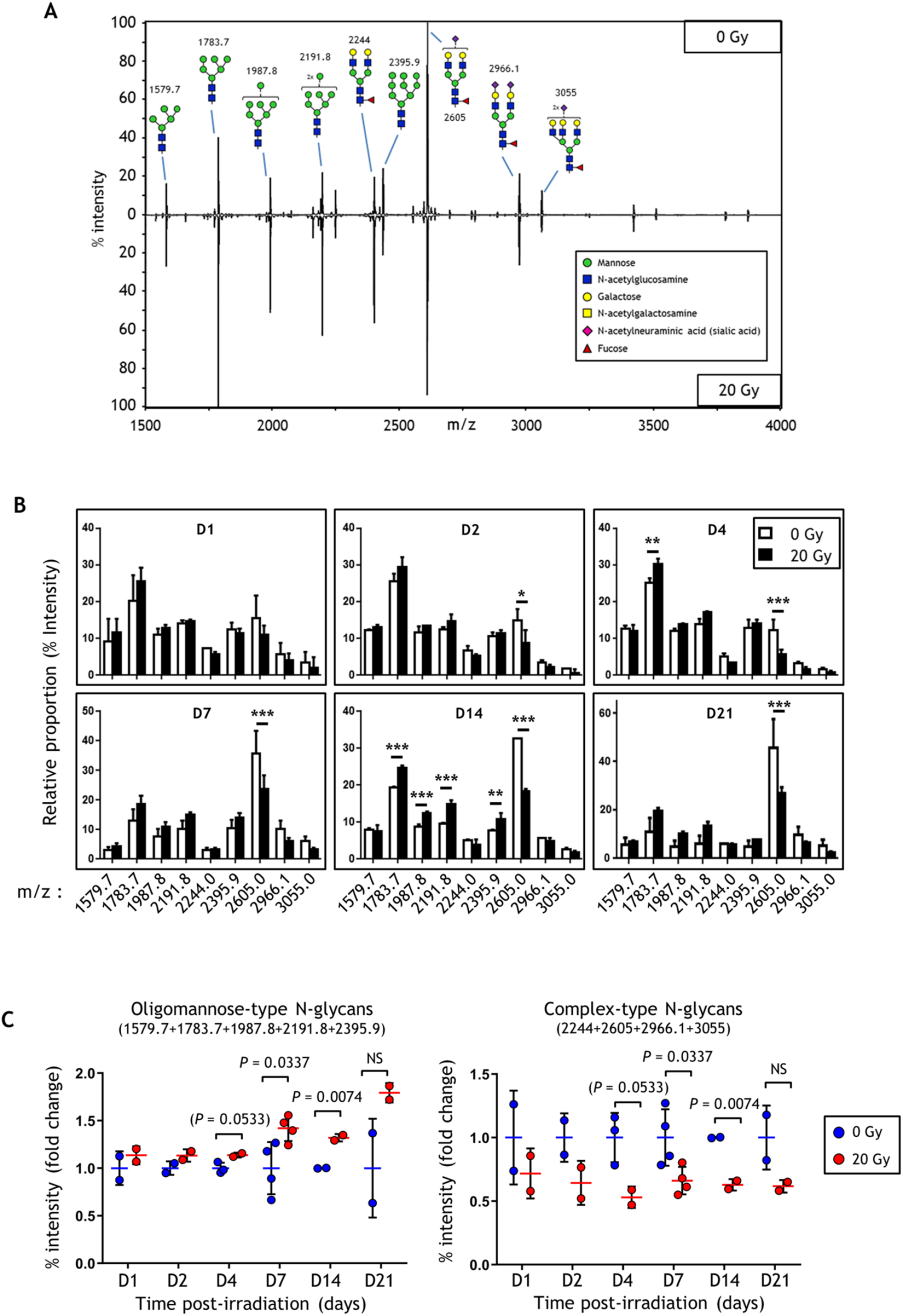
Quantification of N-glycan structures of HUVECs by MALDI-TOF mass spectrometry. (**A**) Representative MALDI-TOF MS mirror spectra of permethylated N*-*glycans derived from control and 20 Gy-irradiated HUVECs 14 days post-exposure. (**B**) Changes in relative abundances of the nine structure species observed in glycosylation patterns between control and 20 Gy-irradiated HUVECs (mean +/− SD). Data were analyzed by two-way ANOVA. *p < 0.05; **p < 0.01; ***p < 0.001. (**C**) Changes in relative abundances of oligomannose- and complex-type N-glycans observed in glycosylation patterns between control and 20 Gy-irradiated HUVECs (mean +/− SD). Data analyzed by two-tailed *t*-test. The experiment was replicated twice (two different cultures) with n = 2 repeated measures and n = 3 spectrum acquisitions within each replication for D7, and twice (two different cultures) with n = 1 repeated measures and n = 3 spectrum acquisitions within each replication for the other time points. Data represent the mean of the two experiments.

To confirm these previous observations, the N-glycome of total protein extracts of HUVECs was investigated by MALDI-TOF MS. We used a comparative glycomic approach that distinguished quantitatively between the N-glycan structures derived from 0 and 20 Gy-irradiated HUVECs. Profiles of permethylated glycans from approximately 3 mg of total protein extracts per sample were recorded for the m/z range 1500–5500 Da using MALDI-TOF MS. Due to the complexity of the MS profile, nine different ions were finally selected as possible glycan species. The nine structures are shown in the Supplementary Fig. S4. Figure 2A illustrates representative glycan map profiles of 0 and 20 Gy-irradiated HUVEC proteins 14 days after exposure, and shows qualitatively similar profiles but different peak intensities between the 2 samples. No structure was found to be unique and specific to a particular dose of irradiation. Among these structures, five species contained at least five mannose residues (referred to as high mannose or oligomannose-type N-glycans; m/z 1579.7, 1783.7, 1987.8, 2191.8 and 2395.9) and four species were bi- (three species; m/z 2244, 2605 and 2966.1) or triantennary (one species; m/z 3055) complex-type N-glycans (referred to as complex-type N-glycans). All complex-type N-glycans contained a fucose residue and no (one species; m/z 2244), one (one species; m/z 2605) or two (two species; m/z 2966.1 and 3055) N-acetylneuraminic acid residues. These results confirmed that HUVECs expressed high mannose and complex N-glycans. It should be noted that no hybrid-type N-glycans were detected and quantified reliably. The mass lists and their corresponding peak intensities (raw data) are given in Supplementary Table S1.

Changes in relative abundances of the nine structure species and the two glycan types (*i.e*. families) observed in glycosylation patterns between control and irradiated HUVECs were further determined by comparing relative proportions and fold change values of expression from day 1 to day 21 post-exposure (Fig. 2B). Supplementary Table S1 displays all MALDI-TOF MS signal % intensities for each m/z value of the nine glycans for each acquisition. Overall, the results show an increased proportion of oligomannose-type structures and a decreased proportion of complex-type N-glycans. We also determined the relative proportion of the two glycan families, *i.e*. oligomannose- (m/z 1579.7, 1783.7, 1987.8, 2191.8 and 2395.9) and complex-type N-glycans (m/z 2244, 2605, 2966.1 and 3055), by summing the proportions of all glycan structures of each family (Fig. 2C). This way of comparing the relative expressions of the two different families of glycans shows that proportions of oligomannose-type N-glycans increased while complex-type N-glycans decreased at day 7 and day 14 in response to ionizing radiation. These results suggest that the level of complex-type N-glycans decreases in response to radiation. To assess the radiation-induced decrease in expression of these structures, we performed fluorescence microscopy and quantified staining of FITC-PHA-L and FITC-UEA-I lectins (Supplementary Fig. S5), which respectively bind to bi- and tetraantennary complex-type N-glycans, and to α1-2-linked fucose present on complex-type N-glycans^24^ (Supplementary Fig. S1B,C). After an increase at day 1 post-irradiation, the results show clearly decreased staining of FITC-PAH-L from day 4 to day 14. Similarly, FITC-UEA-I decreased after irradiation, but less clearly. Altogether, these results show a decrease in complex-type N-glycan structures following irradiation.

### Ionizing radiation decreases the level of glycosaminoglycans in endothelial cells

Pro-inflammatory stimuli such as TNFα induce shedding of glycosaminoglycans (GAGs), thereby decreasing the width and size of the endothelial glycocalyx^14, 25^. This allows greater accessibility of endothelial glycoprotein epitopes, on which circulating leukocytes roll and to which they adhere. In a proposed model, TNFα modulates the pattern of endothelial N-glycosylation in concert with decreased glycocalyx size which in turn mediates higher affinity interactions with circulating monocytes^26^. To examine whether ionizing radiation affects the glycocalyx, we quantified total GAGs of endothelial cells in a time-course experiment from day 1 to day 14 post-exposure. By using a carbazole assay to quantify uronic acid content, we found that the total GAG content was about 2-fold lower in irradiated HUVECs from day 2, like the effect of TNFα for 24 hours (Supplementary Fig. S6). Interestingly, this decrease lasted over time and even at day 14 a non-significant decreased level could be observed. These results suggest a decrease of the width and size of the endothelial glycocalyx in response to ionizing radiation.

### The radiation-induced stimulation of monocyte-endothelial interactions is partly inhibited by a competitive inhibitor of mannose

**Figure 3:**
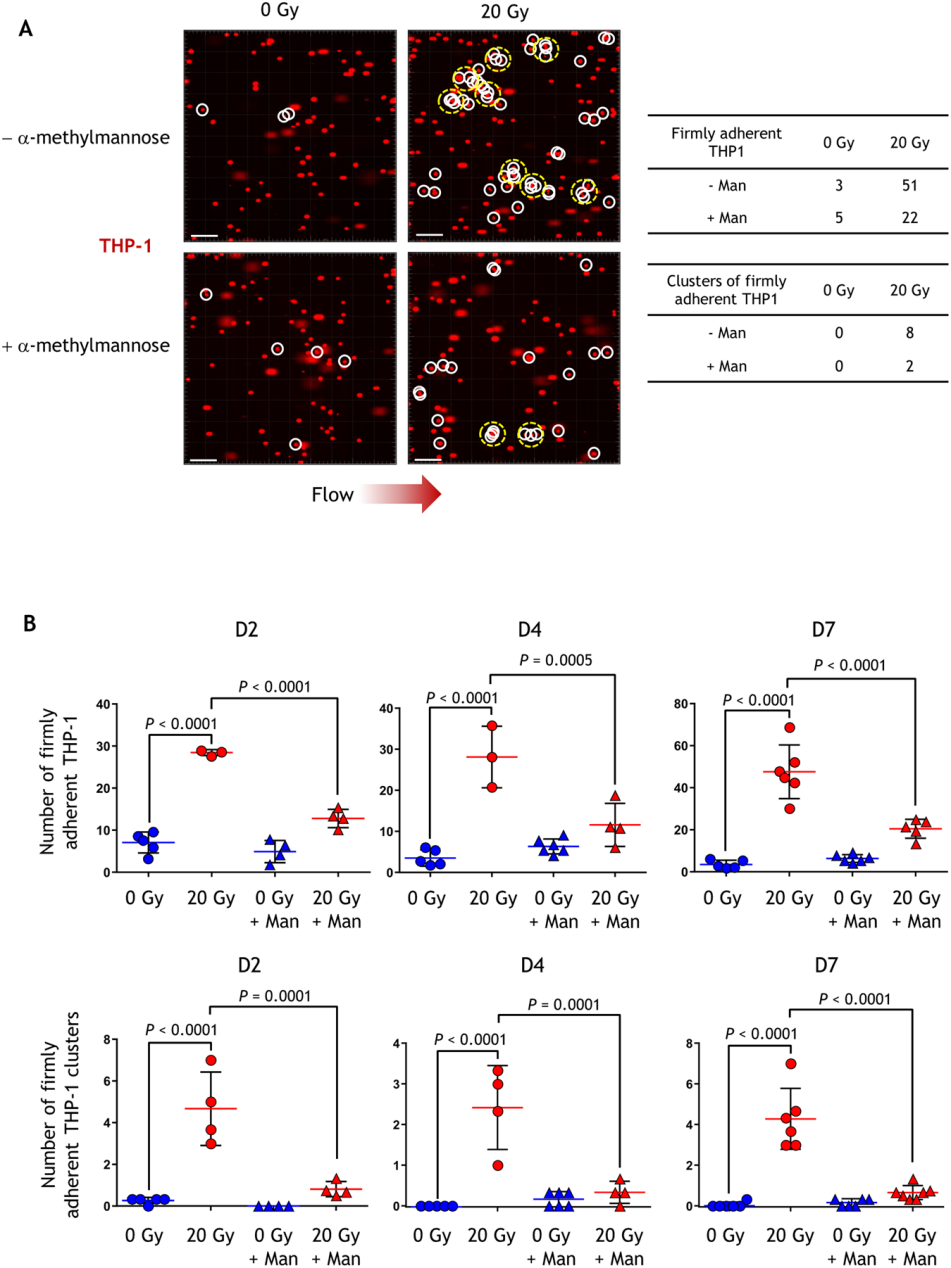
Interaction of THP-1 cells with a monolayer of HUVECs under flow conditions. (**A**) Snapshots of representative movies used in the experiments illustrating the interactions between THP-1 and control or 20 Gy-irradiated HUVECs at day 7 post-irradiation in the presence or absence of the mannose competitive inhibitor α-methylmannose. After 5 minutes of a continuous flow of red-labeled (Cell Tracker) THP-1 cells, a film of ten images was recorded to determine the number of firmly adherent THP1 cells (surrounded by white circles) and the number of clusters of firmly adherent THP-1 cells (three cells or more) (surrounded by yellow dotted-line circles). The number of single and of clusters of firmly adherent THP-1 cells is shown to illustrate this representative example. Scale bars: 100 µm. (**B**) Quantification of firmly adherent THP-1 cells in the presence or absence of α-methylmannose (Man) at different times post-irradiation of HUVECs (mean +/− SD). Firmly adherent THP-1 cells (single and clusters) were counted using the movies recorded in the experiment. The experiment was replicated twice (two different cultures) with at least n = 3 repeated measures from at least three different lamella and with n = 3 repeated measures from three different fields of observation within each lamella, for each time point. Data represent the mean of the two experiments. Data were analyzed by one-way ANOVA.

Since irradiated cells overexpressed high mannose N-glycans and their glycocalyx may have diminished, we wondered whether these modifications could have effects on radiation-induced adhesion of monocytes. Firm adhesions of monocytes were assessed under flow conditions at days 2, 4 and 7 following 20 Gy irradiation. Cell Tracker Red-labeled THP-1 monocytes were continuously flowed for 5 minutes on a confluent monolayer of control or 20 Gy-irradiated endothelial cells, whereupon the number of adherent cells and the number of adherent cell clusters (at least 3 contiguous adherent cells) were counted. We found that the number of firmly adherent monocytes increased dramatically (4- to 10-fold) after irradiation (Fig. 3), as expected based on prior *in vitro* and *in vivo* work^22, 23^. We also found that radiation increased the number of clusters of adherent cells, consistent with the results of a previous study on immortalized coronary artery endothelial cells following irradiation^27^. To test whether increased high mannose N-glycans contribute to radiation-induced adhesion to endothelial cells, a competition experiment was performed in which dynamic flow-dependent THP-1 monocyte adhesion to irradiated HUVECs was evaluated in the absence and presence of α-methylmannose. Figure 3 shows that THP-1 adhesion during flow was affected by α-methylmannose in terms of both the number of adherent THP-1 and the number of clusters of adherent cells. Inhibition was not complete, reaching levels of about 55–60% regarding the number of adherent cells and 80–90% for the number of clusters. This suggests that determinants other than high mannose glycans are likely involved in radiation-induced stimulation of the monocyte-endothelium interaction, possibly through other glycan structures.

### The glycosylation gene expression profile of endothelial cells is deeply modified by ionizing radiation

**Figure 4:**
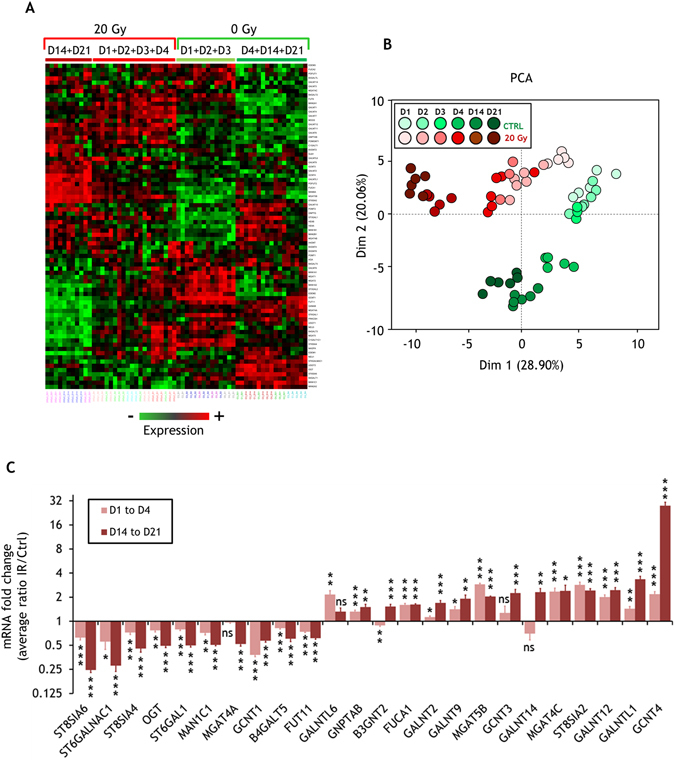
Glycosylation transcriptome analysis of irradiated HUVECs. (**A**) Heat map of the hierarchical clustering of all HUVEC samples based on measured gene expression values. (**B**) Principal component analysis of all HUVEC samples based on measured gene expression values. (**C**) Fold changes of statistically differentially expressed mRNA (24 genes) between control (0 Gy) and 20 Gy-irradiated HUVEC samples (fold change threshold 1.5) (mean +/− SEM, Log_2_ scale). The experiment was replicated twice (two different cultures) with n = 3 repeated measures within the replications for each time point, except for day 3 which was performed once with n = 3 repeated measures. Data represent the mean of the two experiments. Hierarchical clustering and PCA were performed using all samples from the two experiments. The histogram was built from the two experiments and by grouping the data from D1 to D4 (with finally n = 19 for 0 Gy samples and n = 20 for 20 Gy samples), and from D14 to D21 (with finally n = 12 for 0 Gy samples and n = 11 for 20 Gy samples). Data were analyzed by a two-tailed *t*-test. Adjusted *p*-values (Benjamini-Hochberg procedure) (Ctrl vs IR): ns, non-significant (p ≥ 0.05); *p < 0.05; **p < 0.01; ***p < 0.001. Ct, ΔCt, 2^−ΔCt^, fold changes, SD, SEM and *p*-values are displayed in the Supplementary Table S3.

Numerous data highlight the key role of transcriptional regulation control of the enzymes in determining the glycosylation profile, in terms of abundance and diversity, of a cell^28^. In order to gain insight into the mechanism by which irradiation modulates the glycosylation of endothelial cells, and to test whether other modifications of glycosylation could occur, we used quantitative PCR to profile the expression of 84 key human genes encoding enzymes involved in glycosylation (see Supplementary Table S2 for all gene names and abbreviations). Control and 20 Gy-irradiated HUVECs were profiled at early (days 1 to 4) and late (day 14 and 21) times post-irradiation (Fig. 4 and Supplementary Table S3). Hierarchical clustering analysis using the 77 detected and reliably measured genes out of the 84 indicates a clear clustering of control and irradiated samples, whatever the time after exposure (Fig. 4A). A PCA of the dataset (Fig. 4B) and a hierarchical classification on principal components of this PCA (Supplementary Fig. S7A) confirm the differences between the samples on the basis of transcriptional variations of these 77 glycosylation genes, especially at later time points. These results clearly indicate that ionizing radiation modifies the expression of genes involved in glycosylation.

The two different non-supervised multivariate statistical analyses show a clear clustering of early (days 1 to day 4) and late (days 14 and 21) times after irradiation. We therefore grouped data to select genes that were differentially expressed in these two periods. For each time period, we selected genes that were modified by a fold change of at least 1.5 and an adjusted Student’s *t*-test *p*-value less than 0.05 to minimize the false discovery rate, leading to selection of 24 statistically differentially expressed genes at early and/or late time points (Fig. 4C and Supplementary Table S3). Using the dataset obtained from expression measurements of these 24 genes, unsupervised multivariate statistical analyses show a more obvious clustering of control and irradiated samples, and of early and late time points (Supplementary Fig. S7B,C). A time-course analysis of the 24 selected genes shows that these effects were amplified over time (Supplementary Table S3). Examples of such time effects are given in Supplementary Fig. S8 for key genes involved in N-glycosylation (MAN1C1), glycan degradation (FUCA1) and O-glycosylation (GALNTL1 and GCNT4).

### Ionizing radiation stimulates expression of O-glycans in endothelial cells

**Figure 5:**
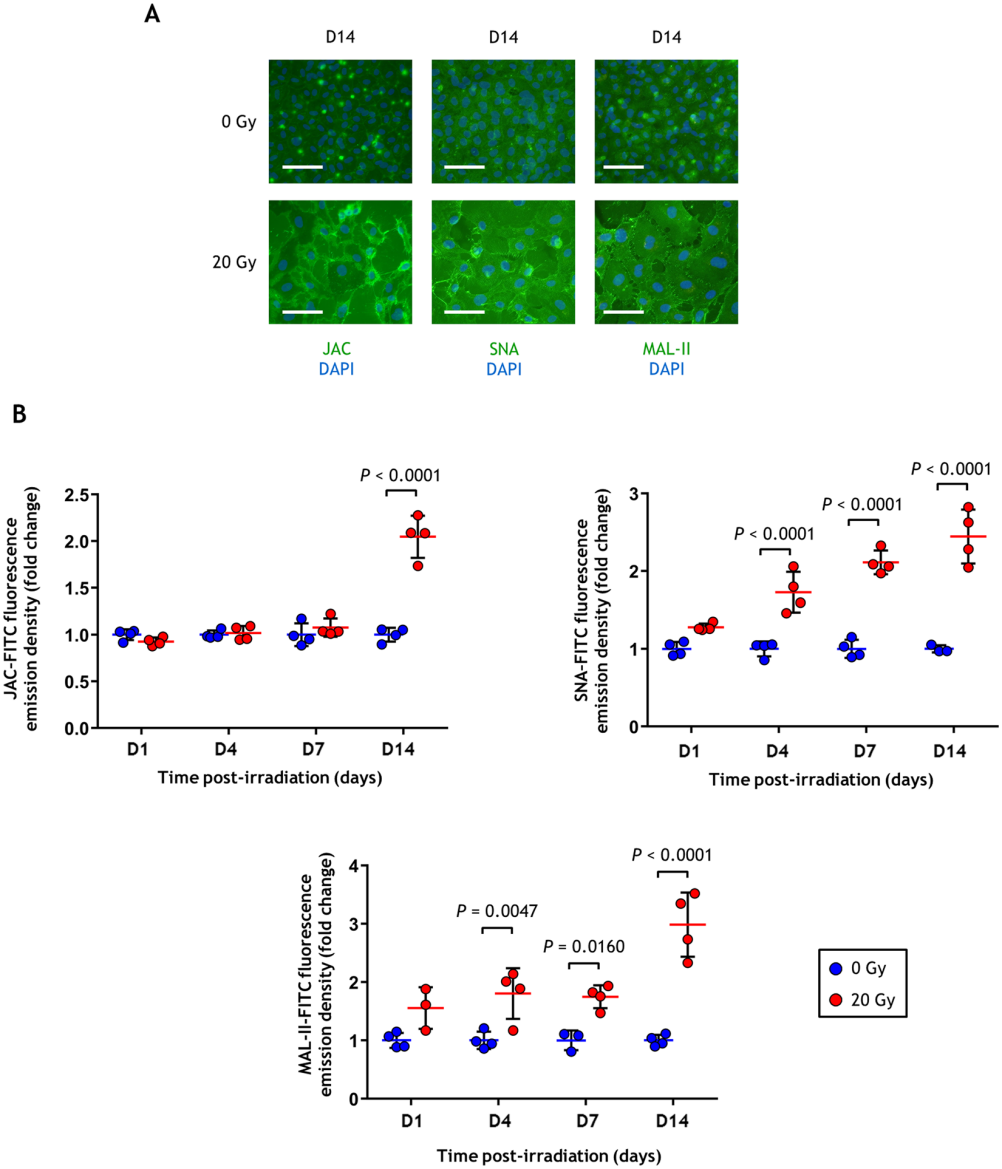
Quantification of JAC, SNA and MAL-II labeling after irradiation. (**A**) Representative fluorescence microscopy images of HUVECs labeled with FITC-JAC, FITC-SNA and FITC-MAL-II. Scale bars: 100 µm. (**B**) Fold changes of average FITC-JAC, FITC-SNA and FITC-MAL-II fluorescence emission densities determined by fluorescence microscopy (mean +/− SD). Experiments were replicated twice (two different cultures) with n = 4 repeated measures within each replication for each time point. Data represent one of the two experiments. Data were analyzed by two-way ANOVA.

Since many genes involved in O-glycosylation were overexpressed after irradiation, we wondered whether irradiated cells display an increase in plasma membrane O-glycans. We performed fluorescence microscopy and quantified the staining of FITC-JAC, FITC-SNA and FITC-MAL-II lectins, the first of which binds to mucins while the second and third bind to N-acetylneuraminic acid through particular carbohydrate structures^24^ (Supplementary Fig. S1D–F). The results show a clear increase of the staining of the three labeled lectins, especially at the last time point (day 14) post-irradiation (Fig. 5). These increases took place from day 4 for SNA and MAL-II. These results are in agreement with the transcriptomic profile and show an increase in O-glycans in response to ionizing radiation.

### *In vivo* localized exposure to radiation induces modifications of glycosylation gene expression pattern in the small intestine

**Figure 6:**
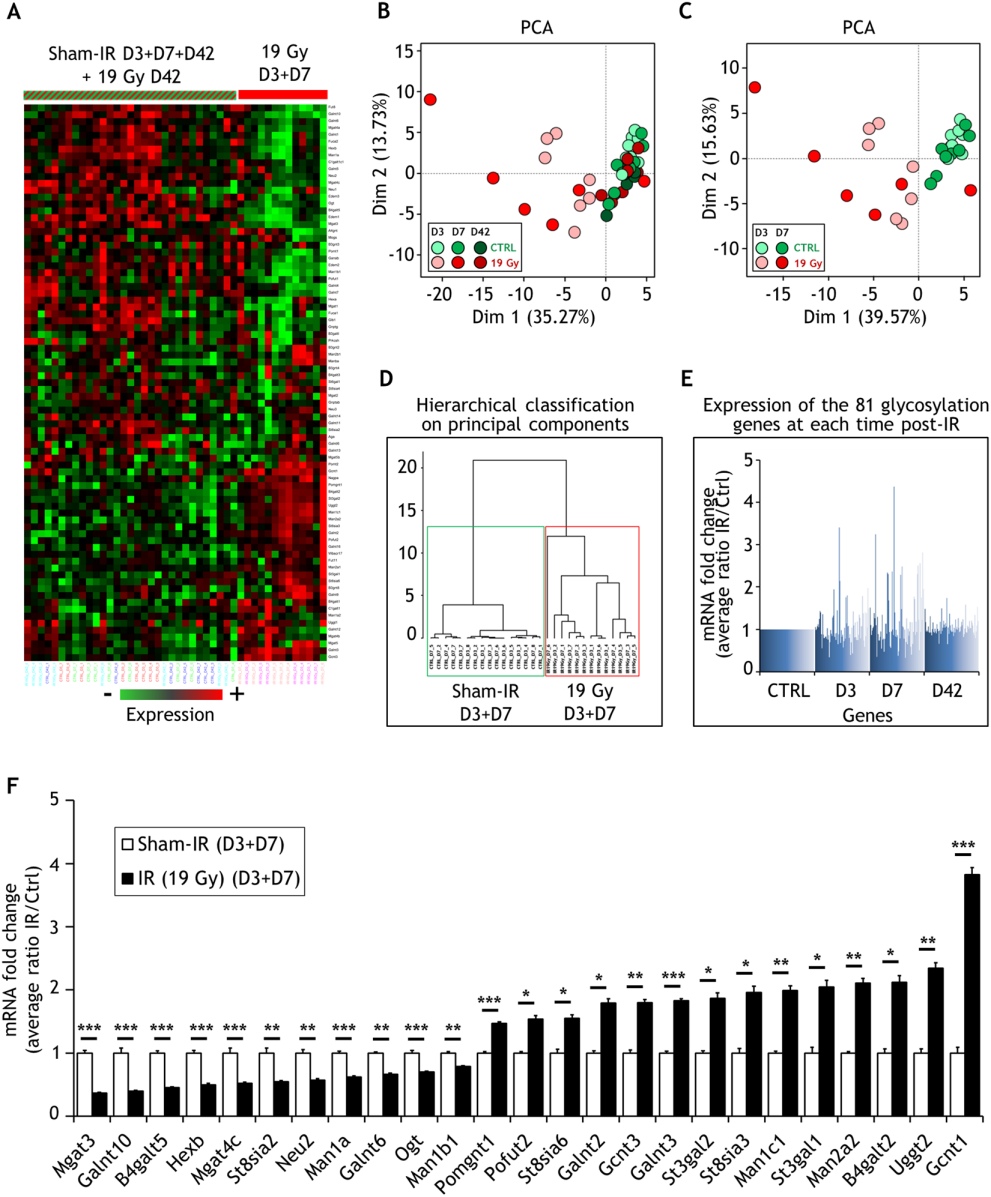
Glycosylation transcriptome analysis of irradiated C57BL/6 small intestine. (**A**) Heat map of the hierarchical clustering of all mouse samples based on measured gene expression values. (**B**) Principal component analysis of all mouse samples based on measured gene expression values. (**C**) Principal component analysis of D3 and D7 mouse samples based on measured gene expression values. (**D**) Hierarchical classification on principal components from the PCA of D3 and D7 mouse samples. (**E**) Fold change expression (sham-IR vs 19 Gy) of the measured glycosylation genes at each time post-IR. (**F**) Fold changes of statistically differentially expressed mRNA (25 genes) between sham- and 19 Gy-irradiated C57BL/6 samples (threshold 1.5 Sham-IR vs 19 Gy at D3 or D7) (mean +/− SEM). The experiment was performed once with n = 8 mice for sham-irradiation at day 3, n = 7 mice for 19 Gy-irradiation at day 3, n = 8 mice for sham-irradiation at day 7, n = 6 mice for 19 Gy-irradiation at day 7, n = 7 mice for sham-irradiation at day 42, and n = 8 mice for 19 Gy-irradiation at day 42. The histogram (F) was built by grouping the data from D3 and D7, with final n = 16 for sham-irradiated mice and n = 13 for 19 Gy-irradiated mice. Data were analyzed by a two-tailed *t*-test. Adjusted *p*-values (Benjamini-Hochberg procedure) (Ctrl vs IR): *p < 0.05; **p < 0.01; ***p < 0.001. Ct, ΔCt, 2^−ΔCt^, fold changes, SD, SEM and *p*-values are displayed in the Supplementary Table S4.

Since ionizing radiation induced dramatic changes in glycosylation gene and glycan expression in endothelial cells, we sought a proof of principle that this profile could also be modified *in vivo*. We assessed the glycosylation gene expression profile in a radiation enteropathy mouse model. In this model, a small part of the intestine was exposed to a high dose of X-rays (19 Gy) to induce acute and late lesions similar to the side effects of radiation therapy^29^. We used the total RNA of the intestine to profile by quantitative PCR the expression of 84 key mouse genes encoding enzymes involved in glycosylation (see Supplementary Table S2 for all gene names and abbreviations). Sham- and 19 Gy-irradiated mice were profiled at days 3, 7 and 42 post-irradiation (Fig. 6 and Supplementary Table S4). Hierarchical clustering and PCA analyses using the 81 detected and reliably measured genes out of the 84 as a dataset indicate that day 3 and day 7 irradiated mice were different from day 3, day 7 and day 42 sham-irradiated mice, and from day 42 irradiated mice (Fig. 6A–D). These results clearly indicate that ionizing radiation modifies the expression of genes involved in glycosylation in the irradiated intestine. As shown in Fig. 6E, the variation of expression occurred mainly at days 3 and 7. We therefore grouped data to select genes that were differentially expressed at days 3 and 7. For each time period, we selected genes that were modified by a fold change of at least 1.5 with an adjusted *p*-value less than 0.05, leading to selection of 25 statistically differentially expressed genes at early time points (Fig. 6F and Supplementary Table S4). As in endothelial cells, many differentially expressed genes encode enzymes involved in N-glycosylation and O-glycosylation.

### Pathway analysis of differentially expressed genes from HUVECs and mouse intestine

**Figure 7:**
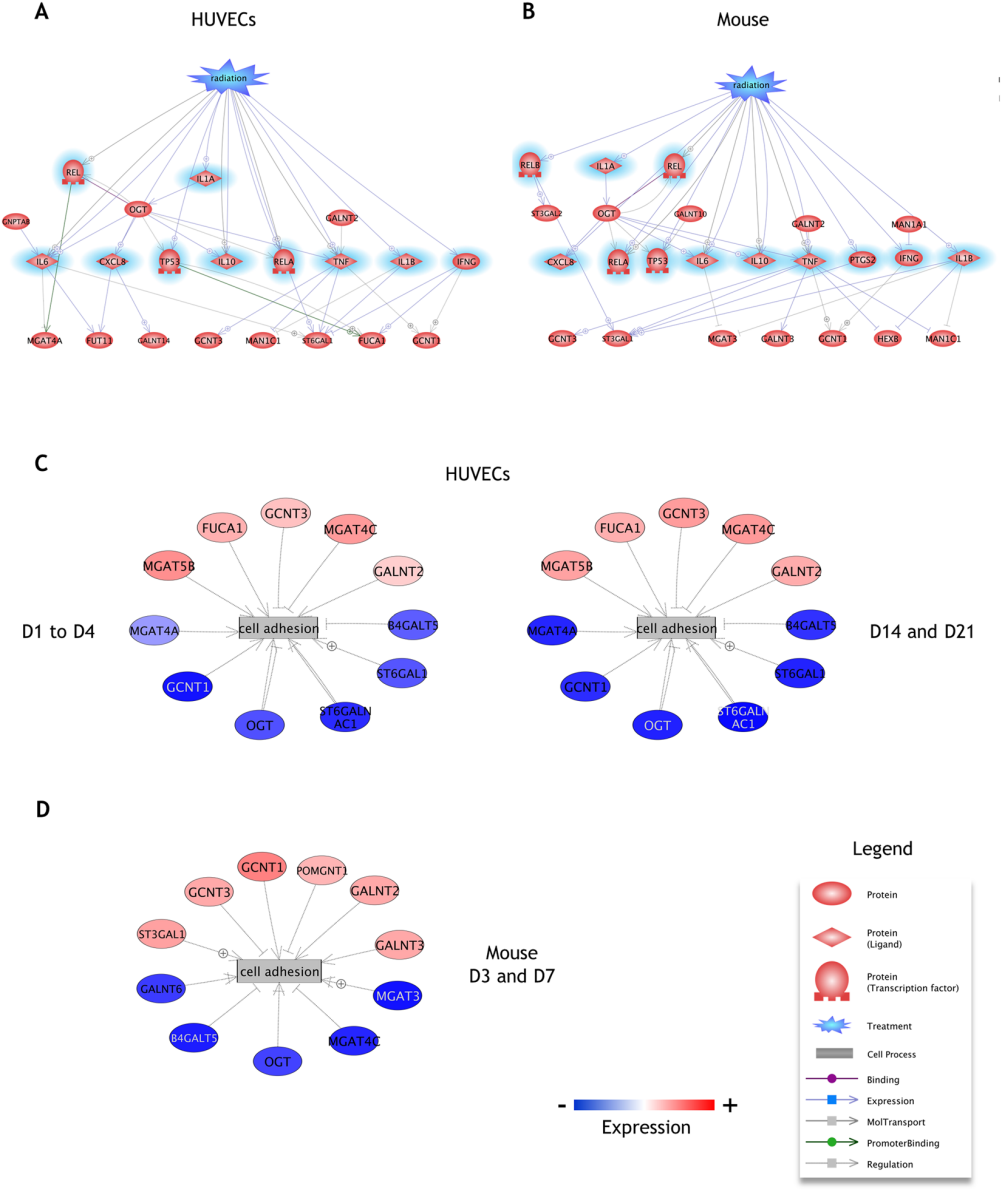
Pathway analysis of statistically differentially expressed genes. (**A**,**B**) Relationships of the 24 selected HUVEC genes and the 25 selected mouse genes to “Radiation” and common genes/proteins (highlighted in blue) involved in the response to ionizing radiation: CXCL8 (MIP-1 in mouse), CXCL12, IFNγ, IL-1α, IL-1β, IL-6, IL-10, NF-κB1, PTGS2, REL, RELA, RELB, TNFα and TP53. (**C**,**D**) Networks of statistically differentially expressed genes linked by the regulating cell process “Cell adhesion”. Genes in blue and red ovals are respectively under- and over-expressed at the indicated days post-irradiation. Relationship details are given in Supplementary Table S5.

Pathway Studio software was employed to establish the molecular interaction and regulation network based on high-throughput interaction datasets. Biological interactions of the selected differentially regulated genes were explored by interpreting high-throughput expression data to identify relationships among the proteins and cellular processes involved, and to organize the data into functional networks based on a repository database of known protein-protein interactions and functional interactions. We first asked whether the selected differentially expressed endothelial and mouse genes shared interactions with 14 genes commonly involved in the regulation of gene transcription induced by irradiation^30–36^. Interestingly, pathway analyses show that 11 out of the 24 differential HUVEC genes share relationships with 10 out of the 14 radiation genes (Fig. 7A), and that 12 out of the 25 differential mouse genes share relationships with 12 out of the 14 radiation genes (Fig. 7B and Supplementary Table S5 for the detailed relationships). We also identified an important gene network around the cell process “cell adhesion” from pathway analysis using the enriched sub-networks plugin of Pathway Studio with which we searched for significant cell processes using HUVEC and mouse differentially expressed genes (Supplementary Table S5). We used these enriched sub-networks to build a pathway in which the genes were connected to the cell adhesion process and colored according to their differential under- or overexpression (Fig. 7C and D and Supplementary Table S5 for the detailed relationships). Among the 24 differential HUVEC genes, 11 genes were found to be related to cell adhesion, indicating that radiation may also affect this cell process through the modulation of expression of these genes. Similarly, 11 genes among the 25 differential mouse genes were found to be related to the cell adhesion process, suggesting that the latter could also be modified in the irradiated intestine.

## Discussion

Vascular endothelium is the primary entry point of immune cells into radiation-injured normal tissues and tumors. While this recruitment can be beneficial in the control of tumor cells^2^, a number of studies suggest that it may become detrimental to healthy tissues when chronic and unresolved^6^, and possibly participates in the initiation and/or the development of acute and late adverse tissue effects in the course of radiation therapy. Controlling this recruitment may thus open up therapeutic opportunities to protect healthy tissues from radiation while increasing tumor control. However, the mechanisms by which radiation sustainably activates endothelial cells are largely unknown. The work presented herein provides new insights into endothelium-monocyte interactions induced by radiation exposure, where high mannose N-glycans and likely other glycans may act as ligands for monocyte adhesion. Radiation modified the gene expression profiles of both human endothelial cells and mouse intestine, which supports observations made on glycan levels. These modifications could partly explain the modification of the glycome, which is mainly controlled by transcriptional regulations^28, 37^. *In silico* analyses of differential genes finally indicate an important pathway around the cell adhesion process. Relationships between common proteins involved in the response to radiation and differential glycosylation genes provide clues to how radiation may directly influence their expression. These pathways show links between some of the differential glycosylation genes and these well-known radiation proteins, which are mainly regulated by radiation-induced release of reactive oxygen species and the DNA damage response. Collectively, these data identify temporal radiation-induced modifications of glycosylation as a novel potential regulator of cell adhesion following radiation exposure.

In this study we used high single doses of ionizing radiation, *i.e*. 20 Gy for HUVECs and 19 Gy for mouse intestine, which may appear to be far from conventionally fractionated radiation therapy protocols that deliver daily dose fractions of 1.8–2 Gy. However, in the mouse, a single dose of 19 Gy induces biological effects similar to the side effects of fractionated radiation therapy^29^. In addition, using this model, we showed the involvement of endothelial cells in the initiation and the development of radiation toxicity in mouse^38^. On the other hand, advances in treatment planning and delivery have made it possible to deliver one or more fractions of high-dose ionizing radiation (15–20 Gy) to tumors by stereotactic body radiation therapy (SBRT)^39^, which is increasingly being used to treat patients^40^. Endothelial cells from both normal tissues and tumors are thus expected to be exposed to single fractions of high radiation doses such as those used in SBRT. In our work, *in vitro* studies in HUVECs show that this high dose of irradiation changed the glycome profile of cells, and especially stimulated sustainable overexpression of high mannose structures. These results are in accordance with MS and lectin staining experiments showing that cells expressed less complex-type N-glycans. Since the biosynthesis of N-glycans is sequential, early processing steps that trim mannoses are absolutely required to allow late processing steps leading to hybrid and complex N-glycans^37^. Supporting this information, gene expression analyses revealed that MAN1C1, a gene encoding a key mannosidase 1 involved in the earliest steps of mannose removal, was down-regulated in response to radiation. This decrease was amplified with time post-irradiation, consistent with the results of Con A binding and MS experiments. These results suggest a sustainable up-regulation of oligomannose-type N-glycans that may be part of the chronic alteration of the endothelial phenotype. Similarly, we found that *in vivo* localized irradiation of mouse intestine also down-regulated the expression at early post-irradiation times of the two mannosidase genes MAN1A1 and MAN1B1, which are also involved in the earliest steps of the trimming of mannoses. Interestingly, our findings share similarities with earlier work on acute and chronic inflammation showing that TNFα treatment of HUVECs increased surface expression of high mannose/hybrid N-glycans, promoting adhesion of monocytes on the treated cell monolayer^26, 41, 42^. In the same way, alteration of endothelial N-glycan profiles by tumor-conditioned media has been proposed as an important mechanism for tumor extravasation^43^. Also, overexpression of high mannose glycans has been observed under chronic inflammatory conditions in human aortic endothelial cells and in atherosclerotic plaques in ApoE^−/−^ mice and human coronary arteries, revealing potential novel effectors of monocyte adhesion during atherogenesis^44^. This increase in high mannose N-glycans, associated with stimulation of monocyte adhesion, could be due to down-regulation of mannosidases 1 since decreases in gene expression or enzyme activity have been shown in these different inflammatory contexts.

Our results also suggest a general increase in O-glycosylation. Expressions of several genes encoding different polypeptide-N-acetylgalactosamine (GalNAc) transferases (the enzyme that links the first N-acetylgalactosamine to the protein) were found to be durably overexpressed in HUVECs (GALNT2, 9, 12, and 14, GALNTL1 and 6) and mouse (GALNT2, 3, 6 and 10). The transfer of the first sugar from UDP-GalNAc directly to serine or threonine in a protein is essential for the biosynthesis of mucins, which are heavily O-glycosylated glycoproteins^24^. An increase in GalNAc transferase genes could thus induce an increase in O-glycan levels. Consistently, these modifications were supported by lectin binding assays on living endothelial cells showing increased staining of JAC, SNA and MAL-II. To go further, gene expression profiles suggest that radiation may stimulate the expression of core 2, in agreement with UEA-I and MAL-II lectin staining, and core 4 O-glycans (overexpression of the N-acetylglucosaminyl transferases GCNT3 and 4 in HUVECs, and of GCNT1 and 3 in mouse). Core 1 and core 2 O-glycans are typically increased *in vitro* and *in vivo* in inflammatory conditions^45^. Core 2 O-glycans are the main support of the sialyl Lewis^X^ epitopes, which bind to the L-selectin of leukocytes in high endothelial venules^15, 46^. Overexpression of sialyl Lewis^X^ motif on endothelial cells may enhance the interaction between endothelium and circulating cells^45^. Although we did not investigate this important sugar motif in our study, a possible increase in α2-3 sialylation as attested by the increase in MAL-II staining after radiation may suggest an increase of sialylation in agreement with an increase of the sialyl Lewis^X^ motif.

In cancer cells, aberrant sialylation favors tumor growth and progression^47^. However, although the idea is attractive, we cannot speculate that in endothelial cells these changes could also promote tumor growth and progression. Among the genes coding for the sialyl transferases whose expression we measured, alpha (2,6) -sialyltransferase (ST8SIA2) appears to be a good candidate to explain the increase in sialylation because its expression was increased 2- to 4-fold in response to radiation (Supplementary Table S3). In accordance with these results, we noted a statistically significant decrease, albeit small, in the expression of the two detected genes Neu1 and Neu3, which encode sialidases (see Supplementary Table S3). This putative increase after irradiation could thus be involved in the residual adhesion of monocytes to irradiated endothelial cells in the presence of α-methylmannose. In this way, our previous findings that radiation induced overexpression of multiantennary N-glycans on serum proteins^16^, which are expected to carry sialyl Lewis^X^ motifs, support the hypothesis that such modifications might represent a feedback response of the hepatic acute phase reaction in an attempt to reduce the cellular inflammatory reaction^18–20^. However, these protein modifications should not have an inhibitory effect on high mannose-mediating adhesion of monocytes since they do not carry high mannose structures, possibly in connection with the fact that tissues never stop recruiting immune cells in severe radiation-induced lesions.

The current work demonstrates that radiation deeply modifies the glycome of primary endothelial cells and has functional effects on radiation-induced monocyte adhesion, and suggests that these changes may have an essential impact on the recruitment of circulating cells in both healthy tissues and tumors. To go further, it would now be interesting to investigate modifications *in vitro* in a tumor context and *in vivo* in both normal tissues and tumors. Understanding the control and molecular basis of immune cell entry processes might be of therapeutic use both to protect normal tissues against radiation-induced injury and to enhance tumor control. High mannose glycoproteins, for instance high mannose ICAM-1 as previously proposed in the case of chronic inflammatory diseases^48^, should now be considered as a new therapeutic target for controlling radiation-induced leukocyte trafficking and endothelial radiation-induced inflammation. Since our results also suggest that sialylation is modified in response to radiation, sialylated endothelial proteins, such as, for example, NCAM-1, SynCAM-1, NPR-2, CD43, CD31 or CD44, would be interesting to investigate as therapeutic targets since they all act as partners of selectins in migration and extravasation processes.

## Methods

### Cells

HUVECs (pooled donors, product code C2519A, lot number 0000465419) from Lonza were grown at 37 °C with 5% CO_2_ in EBM-2 MV medium containing 5% FBS (Lonza). THP-1 cells from ATCC (LGC Standards, ATTC Number TIB-202, lot number 63176297) were grown at 37 °C with 5% CO_2_ in RPMI 1640 medium (ATCC modification), containing 10% FBS (Thermo Fisher Scientific) and 0.05 mM 2-mercaptoethanol. Cells were authenticated by suppliers. All cells test negative for mycoplasma, bacteria, yeast, and fungi, as guaranteed by suppliers.

### Irradiation procedure

Cell irradiation (single dose of 20 Gy) was performed using a ^137^Cs source (IBL 637, CisBio, 1.15 Gy.min^−1^). All irradiations were carried out at 90–100% confluence and the same number of population doublings (passage 3, corresponding to 9–12 population doublings). Radiation enteropathy was induced by exposure of an intestinal segment to a single dose of 19 Gy as previously described^49^. Briefly, 10-week-old male mice (C57BL/6 from Charles River Laboratories) were anesthetized by spontaneous inhalation of isoflurane–N_2_O gas (Abbott GmbH) and, after laparotomy, a 3 cm long intestinal segment was exteriorized and exposed to a single dose of 19 Gy using an Elekta Synergy^®^ Platform delivering 4 MV X-rays at 2.5 Gy.min^−1^. Sham-irradiation (sham-IR) was performed by maintaining the intestinal segment exteriorized without radiation exposure. After radiation exposure or sham-IR, the exposed segment was returned to the abdominal cavity and peritoneum/abdominal muscles and skin were separately closed with interrupted sutures. Experiments were conducted in compliance with legal regulations in France for animal experimentation, and protocols were approved by the national ethics committee for animal experimentation of the Institute for Radiological Protection and Nuclear Safety no. 81 (protocol 15-04).

### Lectin staining

Monolayers of adherent HUVECs were grown to confluence on microscope coverslips. For incubation with cells, FITC-conjugated lectins (Vector Laboratories) were diluted in their respective HEPES buffers as recommended by the supplier. Cells were washed twice using ice-cold PBS containing 1 mM each MgCl_2_ and CaCl_2_ (PBS++) and then stained with 10 µg.mL^−1^ lectin in their respective HEPES buffers for 15 min on ice in the dark. Cells were washed twice with ice-cold PBS++ and fixed using 4% paraformaldehyde in PBS++ for 10 min at room temperature. Fixed cells were washed twice in ice-cold PBS++ and incubated for 10 min at 4 °C in PBS++. Coverslips were mounted for viewing in Vectashield antifade mounting medium with DAPI (Vector Laboratories) to stain DNA. Images were acquired using a Zeiss Axiophot fluorescent microscope. Surface fluorescence was quantified by the software Histolab (Microvision). The density of fluorescence emission (in intensity level per unit area) was used to compare the difference in labeling between control and 20 Gy-irradiated cells. This quantity was calculated by the ratio between the integrated fluorescence emission and the squared cell surface area of cells.

### Cell tracker labeling of THP-1 monocytes

For adhesion experiments under flow conditions, THP-1 monocytes (1 × 10^6^ cells.mL^−1^) were labeled with 2 µM CellTracker^TM^ Red CMTPX (Thermo Fisher Scientific) in RPMI 1640 without FBS for 45 min at 37 °C in the dark. After centrifugation (120 × g for 6 min), cells were resuspended in RPMI 1640 without FBS and incubated for 30 min at 37 °C in the dark to remove unincorporated dye. Cells were centrifuged (120 × g for 6 min) and resuspended in EBM-2 MV medium without FBS at a concentration of 1 × 10^6^ cells.mL^−1^ and then used in an adhesion assay.

### Sugar inhibition experiments

α-Methylmannose (Vector Laboratories) (200 mM) was mixed with FITC-Con A prior to lectin HUVEC staining and viewing by fluorescence microscopy, or with THP-1 prior to initiation of flow for adhesion experiments.

### Flow cytometry

After dissociation with trypsin, cells (1 × 10^6^) were resuspended in 1 mL of PBS++ containing 5% FBS and stained with To-Pro-3 (Thermo Fisher Scientific) and 10 µg.mL^−1^ FITC-Con A lectin for 15 min on ice in the dark. Multiparametric analyses were performed on BD FACSCanto^TM^ II for data recording and using FlowJo 7.6.5 software (FlowJo LLC) for the analysis of samples. To-Pro-3 fluorescence was collected on the APC channel to record living cell events. For these gated events, gating was done on size (FSC, forward scatter)/granulometry (SSC, side scatter) parameters. Finally, the FITC-Con A signal was collected on the FITC channel. The FSC/FITC-Con A biparametric analyses are reported as dot plot representations. Results are presented as average median values of FITC-Con A fluorescence with at least 1 × 10^4^ gated events per replica. All acquisitions and analyses were performed without compensation as FITC and To-Pro-3 do not overlap.

### MALDI-TOF MS analysis of permethylated N-glycans

HUVECs were washed twice with PBS, scraped, pelleted and stored at −80 °C until N-glycan extraction and analysis. After thawing on ice, cells were sonicated in extraction buffer (25 mM Tris, 150 mM NaCl, 5 mM EDTA and 1% CHAPS, pH 7.4) and then dialyzed in 6–8 kDa cut-off dialysis tubing in an ammonium bicarbonate solution (50 mM, pH 8.3) for 48 hours at 4 °C and lyophilized. About 3 mg of proteins/glycoproteins per sample was reduced and carboxyamidomethylated followed by sequential tryptic and peptide N-glycosidase F digestion and Sep-Pak purification as previously described^50^. Permethylation of the freeze-dried glycans and MALDI-TOF mass spectrometry (MS) of permethylated glycans were performed as described elsewhere^50^. Spectra were obtained by accumulation of 500 laser shots over a range of m/z 1500–5500 Da. Each sample was spotted three times on the MALDI plate and one spectrum was acquired per deposited spot.

### Data evaluation and statistical analysis of mass spectra

The MS profiles of permethylated N-glycans were processed using Data Explorer version 4.7 to generate files containing m/z values and signal intensities after filtering peaks based on a signal-to-noise (S/N) ratio threshold of 3 and a noise filtering/smoothing function (noise removal method, standard deviation to remove: 2). For each m/z value, the peak intensities of the three acquired spectra per replica were averaged prior to further analysis. Furthermore, the processing method took into account isotopic distributions to recalculate S/N ratios to eliminate those under 10. Ions were selected as possible glycan species according to the following criteria: good reproducibility of the signal between the three acquired spectra per sample and between samples (no or few null values), credible structures determined on the basis of composition and biosynthetic pathway knowledge, and S/N ratios over 10. Each monoisotopic signal was assigned to a monosaccharide composition, determined on the basis of composition and biosynthetic pathway knowledge and further classified in two different families: high mannose and complex species. The relative proportion (% intensity) of each structure or family was calculated as the (intensity × 100)/(sum of intensities).

### Cell adhesion assay under flow conditions

Five days after seeding on a glass lamella, HUVECs at confluency were irradiated at 20 Gy. At different times post-irradiation, the lamella was placed in a flow chamber characterized previously^23^ for videomicroscopy analysis. THP-1 monocytes fluorescently labeled with CellTracker^TM^ Red CMPTX were perfused through the flow chamber for a total time of 5 min at a constant wall shear rate of 50 s^−1^ and a constant flow rate of 100 µL.min^−1^ using a syringe coupled to an electric pump (Harvard Apparatus). The entire experiment was visualized in real time at 20× magnification with a video camera (Rolera EM-C² camera, QImaging). Monocyte movements were recorded using MetaVue software (Molecular Devices). The full time-lapse video recorded the movement of monocytes over a 1-min period using a frame rate of 30 frames per second with an exposure of 20 ms per frame. After 5 min of perfusion, a ten-image time-lapse video was recorded to determine the number of firmly adherent monocytes. The time-lapse videos were subsequently analyzed using image analysis IMARIS software version 8 (Bitplane) to determine the number of firmly adherent monocytes.

### RNA isolation, reverse transcription, and real-time quantitative PCR

Total RNA was prepared from HUVECs and mouse intestinal tissue with the total RNA isolation kit (RNeasy Mini Kit, Qiagen), with an additional step of genomic DNA removal by digestion with DNase I (DNA-free^TM^ DNA Removal Kit, Thermo Fisher Scientific). Reverse transcription was performed with 1 µg RNA using the High Capacity Reverse Transcription Kit from Applied Biosystems. Quantitative PCR was carried out on a 7900HT Fast-Real Time PCR system (Applied Biosystems) using the Human or Mouse Glycosylation RT² Profiler^TM^ PCR Arrays (SAbiosciences) to profile the expression of 84 key genes encoding proteins involved in glycosylation. Analyses were conducted according to the procedure previously described in detail^38^. Data Assist software (Applied Biosytems) was used to determine fold changes, with fixed criteria: a maximum allowable Ct value at 35 was fixed and maximum Ct values were not included in calculations. Normalization was performed using a global normalization method on a per sample basis^51^.

### Statistical analyses

Data are given as means ± SD (n < 10) or means ± SEM (n ≥ 10). Statistical analyses were performed by one- or two-way ANOVA, or a two-tailed *t*-test when appropriate, with a level of significance of *p* < 0.05, using GraphPad Prism 7 software. For targeted transcriptomic analyses, Student’s *t*-test *p*-values were adjusted using the Benjamini-Hochberg false discovery rate method using Data Assist software, and a fold change cut-off of 1.5 and an adjusted *p*-value less than 0.05 were applied to select statistically differentially expressed genes. For unsupervised hierarchical clustering analyses and heat map creation with Data Assist software, distances between samples were calculated for hierarchical clustering based on the ΔCT values using Pearson’s correlation, assay centric as map type, and average linkage as clustering method. Principal component analysis (PCA) and hierarchical clustering on principal components were performed with R software^52^.

### Pathway analysis of differentially expressed genes

Pathway and sub-network enrichment analyses were performed using the web version of the software Pathway Studio (Mammalian, ChemEffect, DiseaseFX, version 11.2.5.9, updated Oct 22, 2016) from Elsevier^53^. Names and expression ratio values of the differentially expressed genes were imported into the Pathway Studio. The data input was queried against the Pathway Studio knowledge base for biological interactions. Proteins mapped to the knowledge base were used to build protein networks. Interaction networks were added that included selected neighbors or cell processes.

## Electronic supplementary material

Supplementary Information

Supplementary Table S1

Supplementary Table S2

Supplementary Table S3

Supplementary Table S4

Supplementary Table S5
