# Supplementary material for: Radiation-induced changes in the glycome of endothelial cells with functional consequences
Source: Sci Rep. 2017 Jul 13;7:5290. doi: 10.1038/s41598-017-05563-y (PMC5509684; doi:10.1038/s41598-017-05563-y)
Supplement: Supplementary file 1 — Supplementary Information [file 41598_2017_5563_MOESM1_ESM.pdf]

Radiation-induced changes in the glycome of endothelial cells with functional consequences

Cyprien Jaillet, Willy Morelle, Marie-Christine Slomianny, Vincent Paget, Georges Tarlet, Valérie Buard, Sonia Selbonne, Fanny Caffin, Emilie Rannou, Pierre Martinez, Agnès François, François Foulquier, Fabrice Allain, Fabien Milliat and Olivier Guipaud

Supplementary Information

Supplementary Methods

Phalloidin immunostaining. 20 Gy-irradiated HUVECs were stained with FITC-Con A as above. Cells were either fixed using 4% paraformaldehyde and permeabilized in 0.1% Triton X-100 (Sigma) in PBS (5 min at room temperature) prior to phalloidin immunostaining, or directly immunostained for phalloidin prior to fixation, without membrane permeabilization, to test the plasma membrane integrity of irradiated cells. After washing, CytoPainter F-actin staining kit-Red fluorescence (Abcam) was used according to the manufacturer's instructions to visualize F-actin by the staining of phalloidin. Coverslips were mounted for viewing in Vectashield antifade mounting medium with DAPI.

GAG quantification. Control (0 Gy), 20 Gy-irradiated HUVECs and HUVECs treated for 24 hours with 10 $ng.mL^{-1}$ TNFα (R&D systems) were washed twice with PBS, scraped, pelleted and stored at -80°C until GAG extraction. After thawing on ice, cells were suspended in 1 mL of lysis buffer (50 mM Tris–HCl, pH 7.4, 100 mM NaCl, 1% Triton X-100) and GAGs were prepared as previously described[1] from about 5 mg of proteins per sample. GAGs were quantified using a carbazole assay, according to the method developed by Cesaretti et al.[2] with the previously described modifications[1].

Supplementary References

1. Martinez, P. *et al.* Macrophage polarization alters the expression and sulfation pattern of glycosaminoglycans. *Glycobiology* **25,** 502–513 (2015).
2. Cesaretti, M., Luppi, E., Maccari, F. & Volpi, N. A 96-well assay for uronic acid carbazole reaction. *Carbohydr. Polym.* **54,** 59–61 (2003).

Supplementary Figures

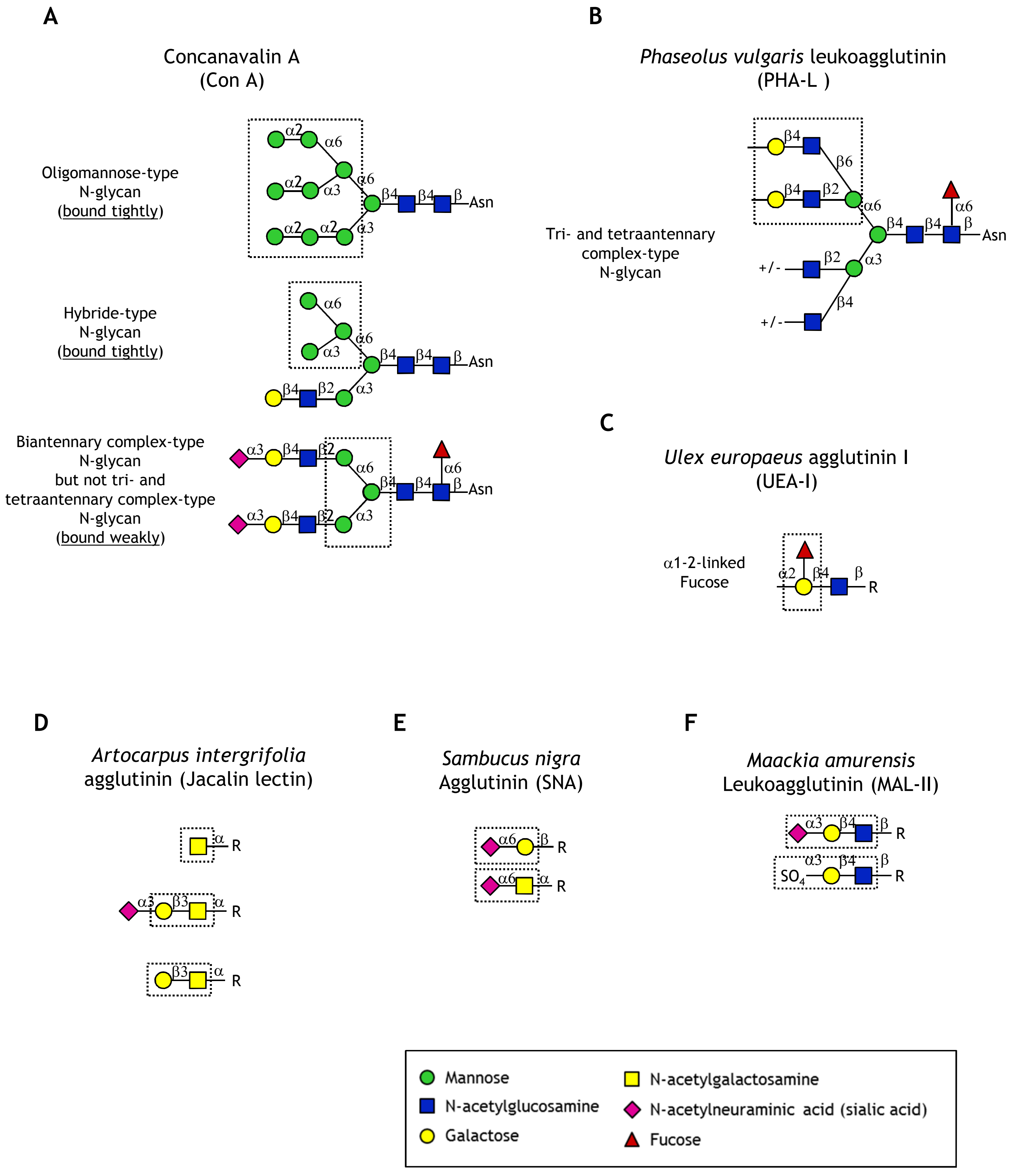

Supplementary Figure S1. Types of glycans determinants bound with high affinity by the different lectins used in the study (from Varki et al, 2009) (24). (**A**) Examples of N-glycans recognized by concanavalin A (Con A). (**B**) N-glycans recognized by *Phaseolus vulgaris* leukoagglutinin (PHA-L). (**C**) Types of glycan recognized by *Ulex europaeus* agglutinin I (UEA-I). (**D**) Types of glycan recognized by *Artocarpus intergrifolia* agglutinin (Jacalin lectin, JAC). (**E**) Types of glycan recognized by *Sambucus nigra* agglutinin (SNA). (**F**) Types of glycan recognized by *Maackia amurensis* leukoagglutinin (MAL-II). The determinants required for binding are indicated in the dotted boxed areas.

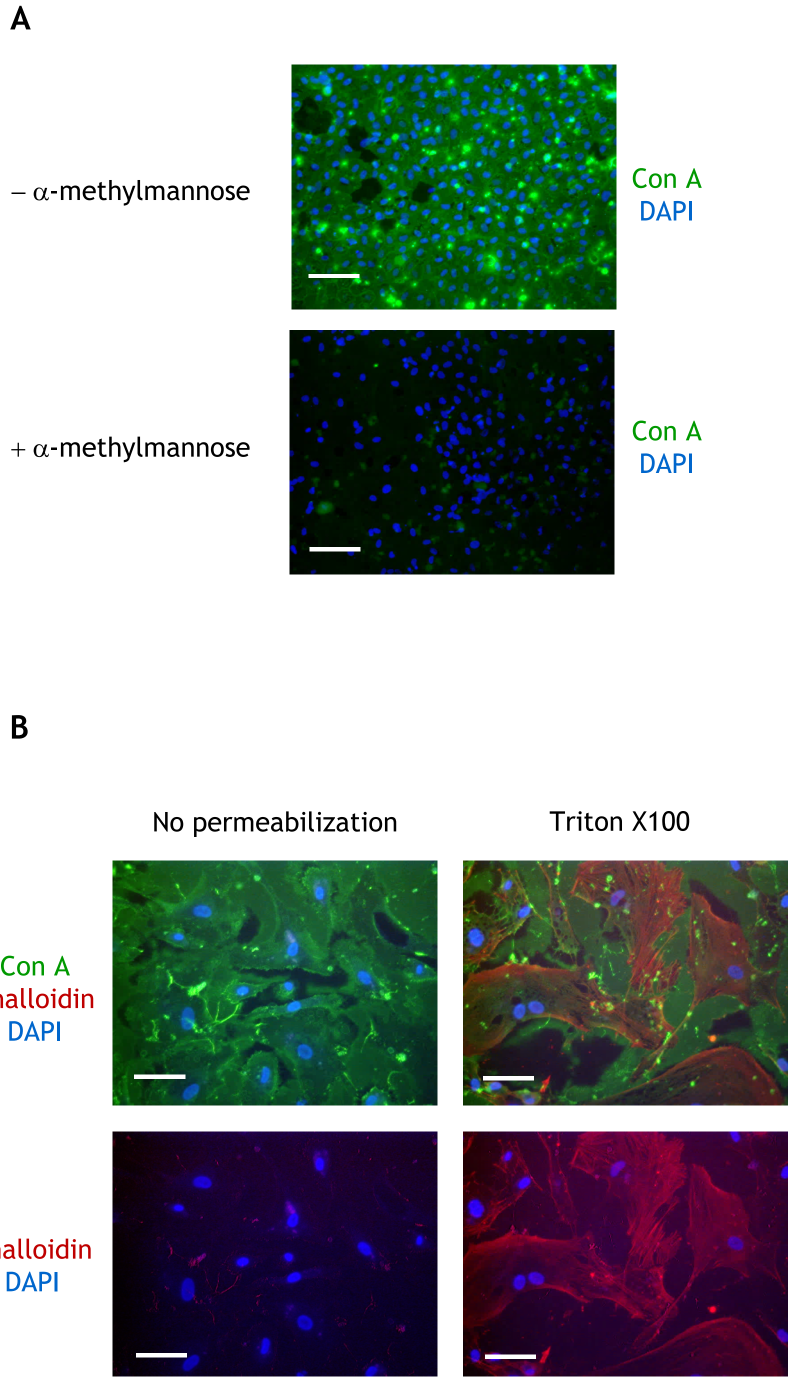

Supplementary Figure S2. High mannose/hybrid N-glycans labeling of the outer surface of the plasma membrane of HUVECs using FITC-Con A. (**A**) Con A staining of non-irradiated HUVECs in the absence and presence of α-methylmannose. Experiment was replicated twice (two different cultures) with n=2 repeated measures within each replication. (**B**) Con A and phalloidin staining of 20 Gy-irradiated HUVECs at day 15 post-exposure with or without membrane permeabilization using Triton X100 prior to staining. The experiment was performed once with n=3 repeated measures. Scale bars: 100 µm.

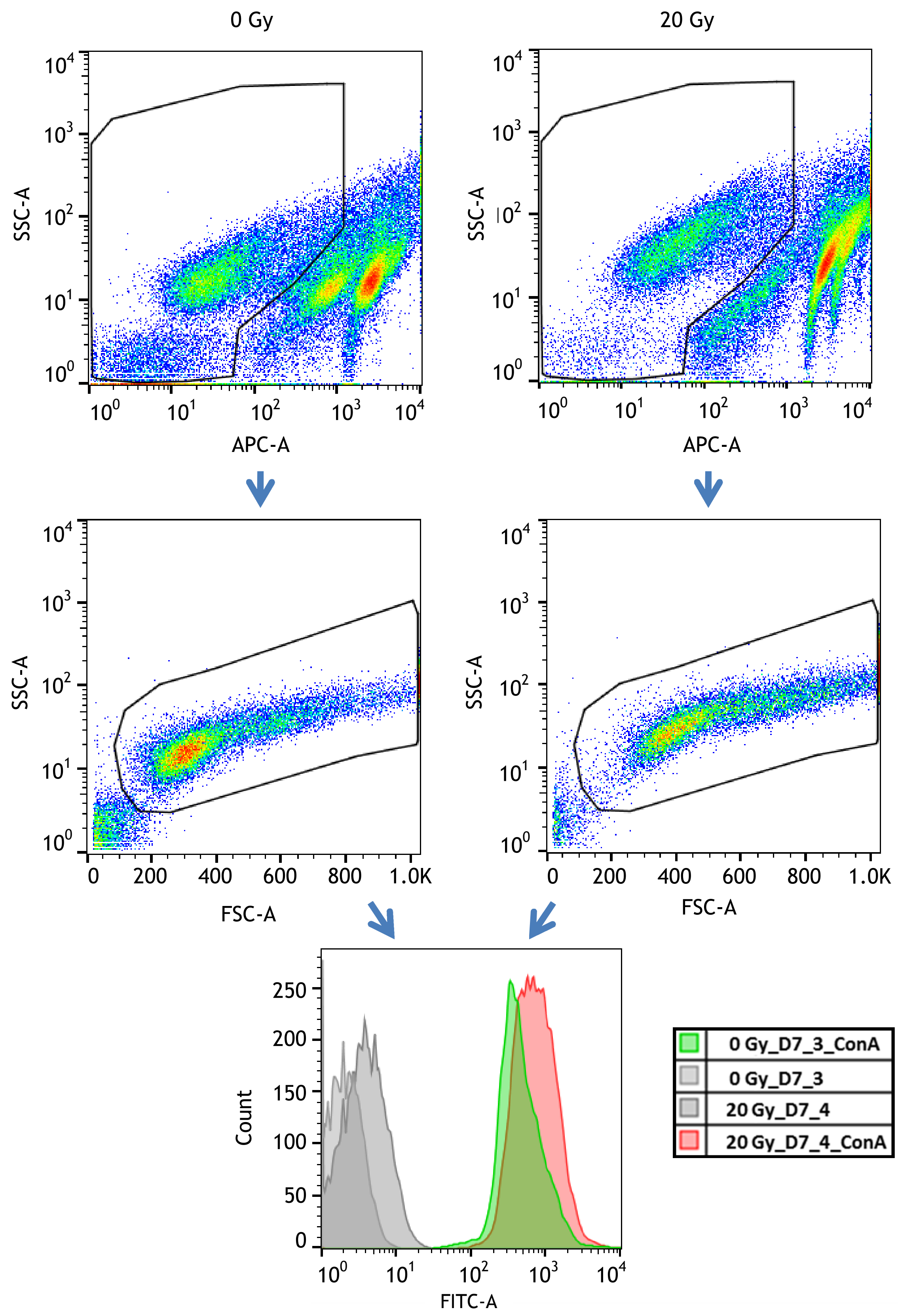

	Sample Name	Count	Median : FITC-A	Mean : FITC-A	Geometric Mean : FITC-A	Count	Median : FSC-A
	0 Gy_D7_3_ConA	16002	417	596	451	16002	336
	0 Gy_D7_3	27946	1,01	1,76	1,50	27946	324
	20 Gy_D7_4	24484	3,21	4,06	3,02	24484	454
	20 Gy_D7_4_ConA	12908	685	869	695	12908	476

Supplementary Figure S3. Gating strategy for flow cytometry analysis. An example of representative FITC-Con A labelled control and 20 Gy-irradiated HUVECs 7 days post-exposure is shown. Cells were first gated on the basis of their viability assessed by the To-Pro-3 labelling (APC-A) (top panels), and then on the basis of their size (FSC-A) and granulometry (SSC-A) (middle panels). The bottom panel shows a merge of the control and 20 Gy-irradiated FITC-Con A labelled cell distributions (the non-labelled cell distributions are also shown). The Table displays the median values of the FITC intensities of the corresponding distributions. SSC, side scatter size; FSC, forward scatter; APC, To-Pro-3; FITC, Con A.

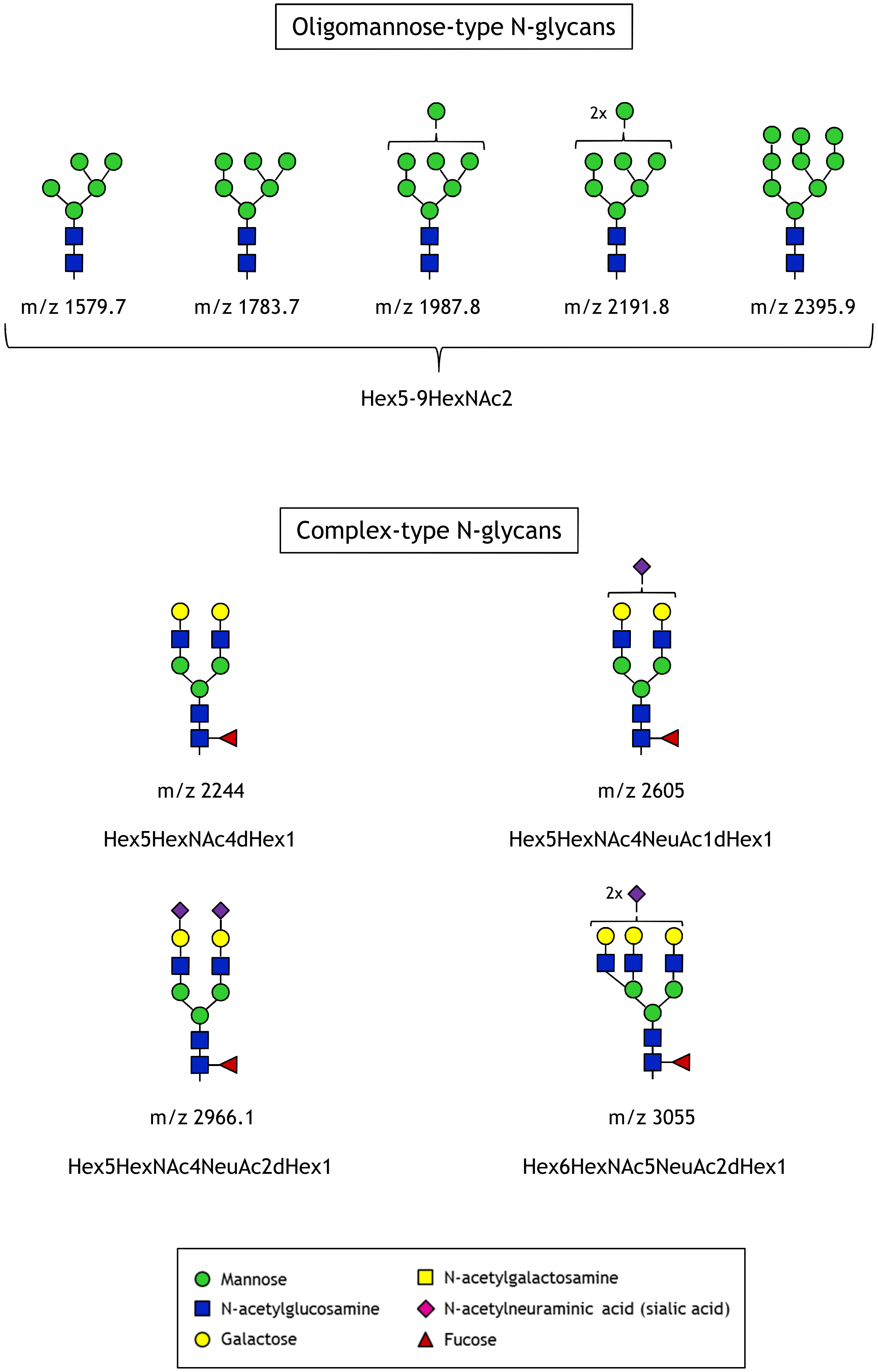

Supplementary Figure S4. m/z and putative structures of the nine N-glycans quantified in the study by MALDI-TOF MS. Putative structures were determined on the basis of composition and biosynthetic pathway knowledge. The structures have been grouped into two types of N-glycans : oligomannose- and complex-type N-glycans.

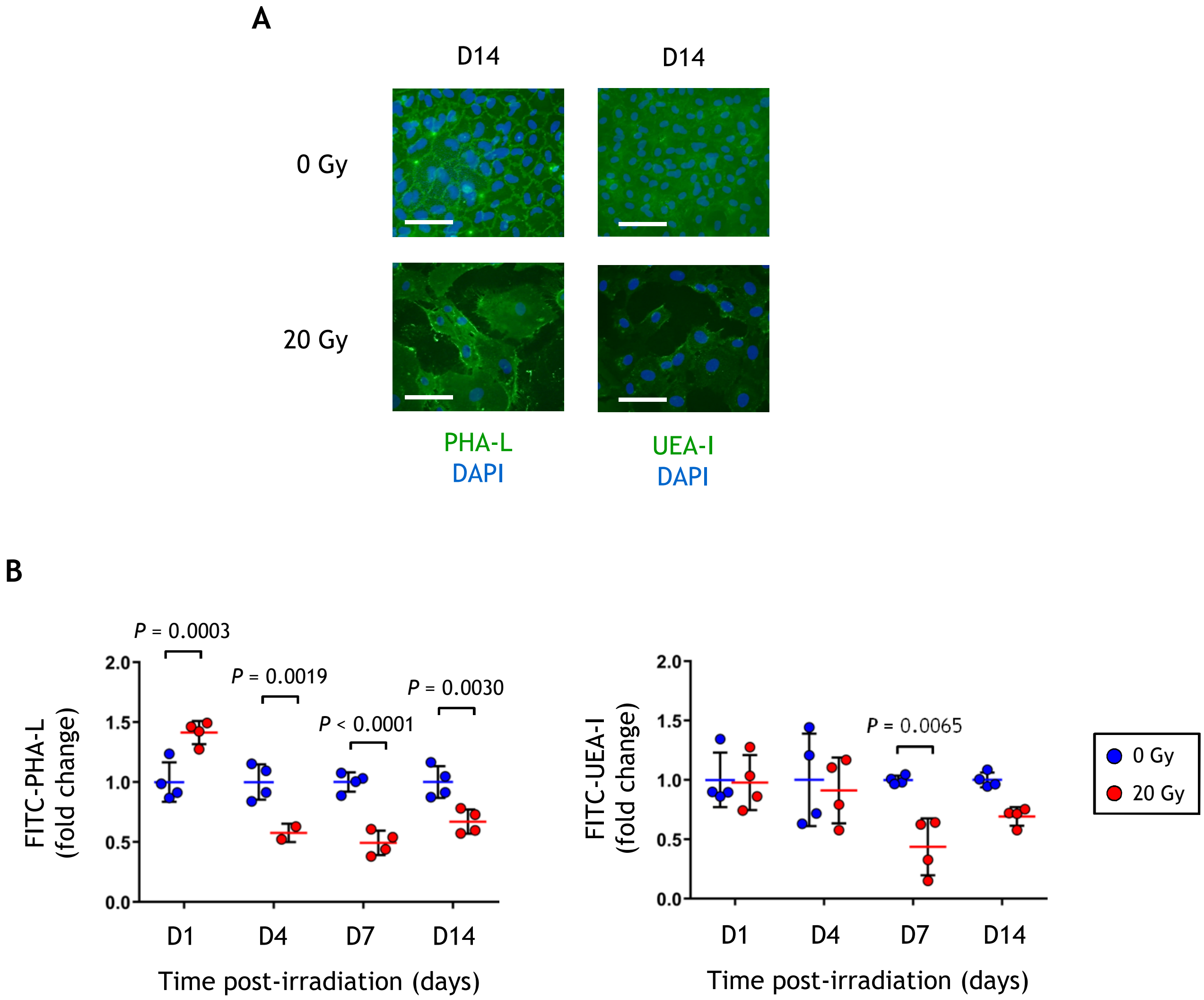

Supplementary Figure S5. Quantification of PHA-L and UEA-I labelling of HUVECs after irradiation (**A**) Representative fluorescence microscopy images of HUVECs labelled with FITC-PHA-L and FITC-UEA-I. Scale bars: 100 µm. (**B**) Fold changes of FITC-PHA-L and FITC-UEA-I fluorescence emission densities determined by fluorescence microscopy (mean +/- SD). Experiment was replicated twice (two different cultures) with n=4 repeated measures within each replication for each time point. Data represent one of the two experiments. Data analyzed by two-way ANOVA .

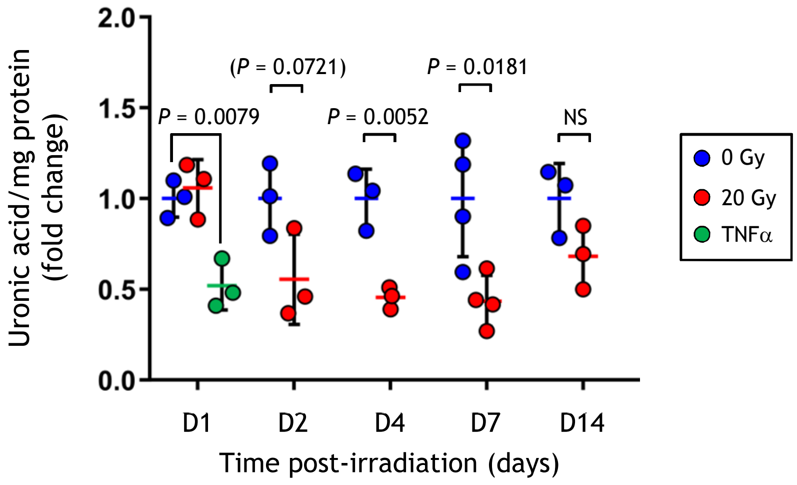

Supplementary Figure S6. Measurements of glycosaminoglycans content in HUVECs after irradiation or TNFα treatment. Fold changes in abundances of uronic acid between control, TNF-α-treated and 20 Gy-irradiated HUVECs (mean +/- SD). Values were normalized by the amount of proteins of the samples after the measurements. Experiment was made once with n=3 repeated measures for D1, D2 and D14, twice (two different cultures) with n=4 repeated measures within each replication for D7, and in triplicate (three different cultures) with n=3 repeated measures within each replication for D4. Data represent one of the experiments. Data analyzed by two-tailed *t*-test.

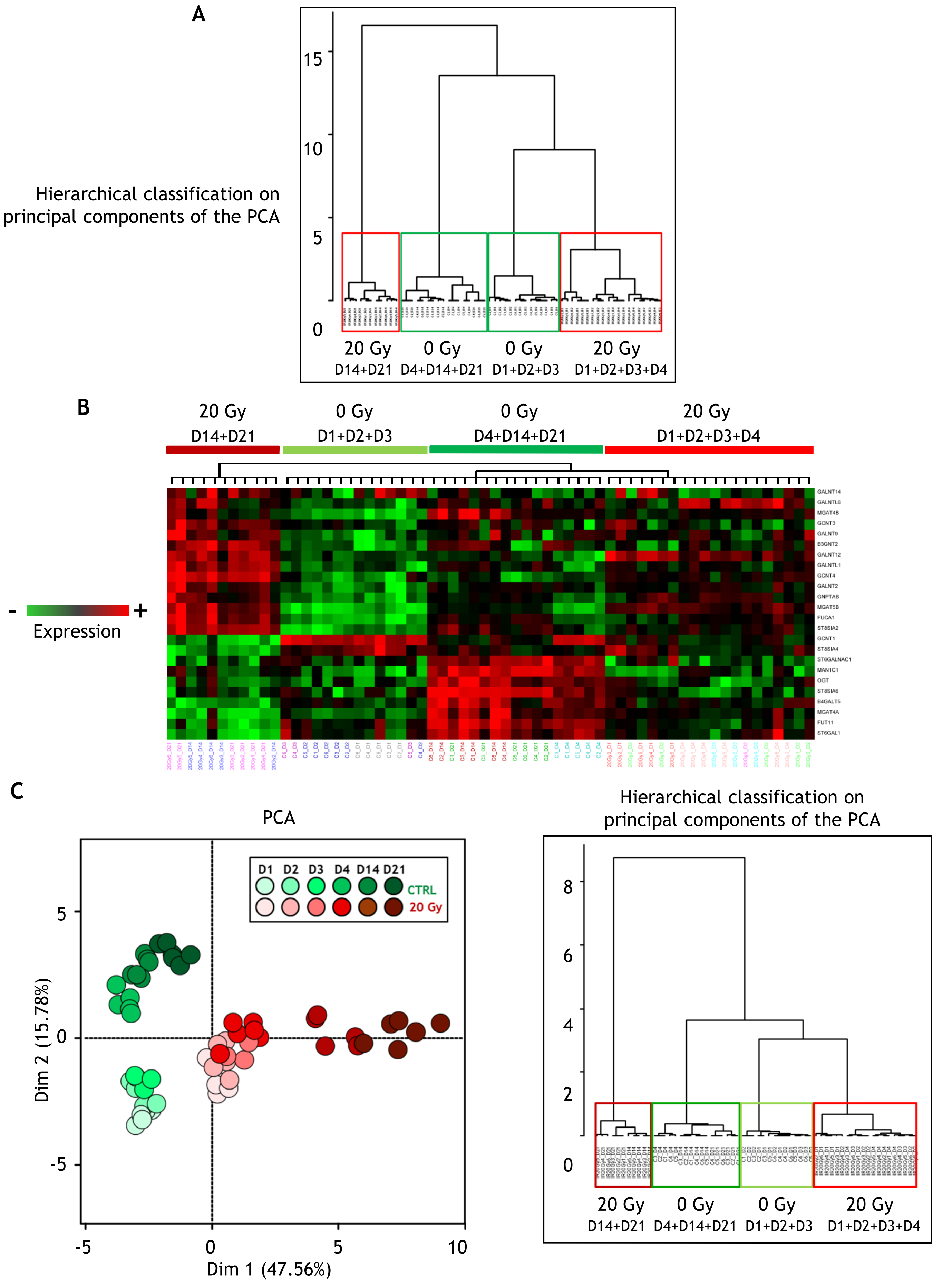

Supplementary Figure S7. Multivariate unsupervised statistical analysis of the targeted transcriptomics dataset from control (0 Gy) and 20 Gy-irradiated HUVECs. (**A**) Hierarchical classification on principal components of the analysis presented in the Fig. 4B (all measured genes as variables). (**B**) Heat map from a hierarchical clustering using the 24 identified statistically differentially genes presented in the Fig. 4C. (**C**) PCA and hierarchical classification on principal components using the 24 differentially identified genes presented in Fig. 4C. The experiment was replicated twice (two different cultures) with n=3 repeated measures within the replications for each time point, except for day 3 which was performed once with n=3 repeated measures. The hierarchical clustering and the PCA were performed using all samples from the two experiments. Ct, ΔCt, $2^{-\Delta Ct}$, fold changes, SD, SEM and *p*-values are displayed in the Supplementary Table S3.

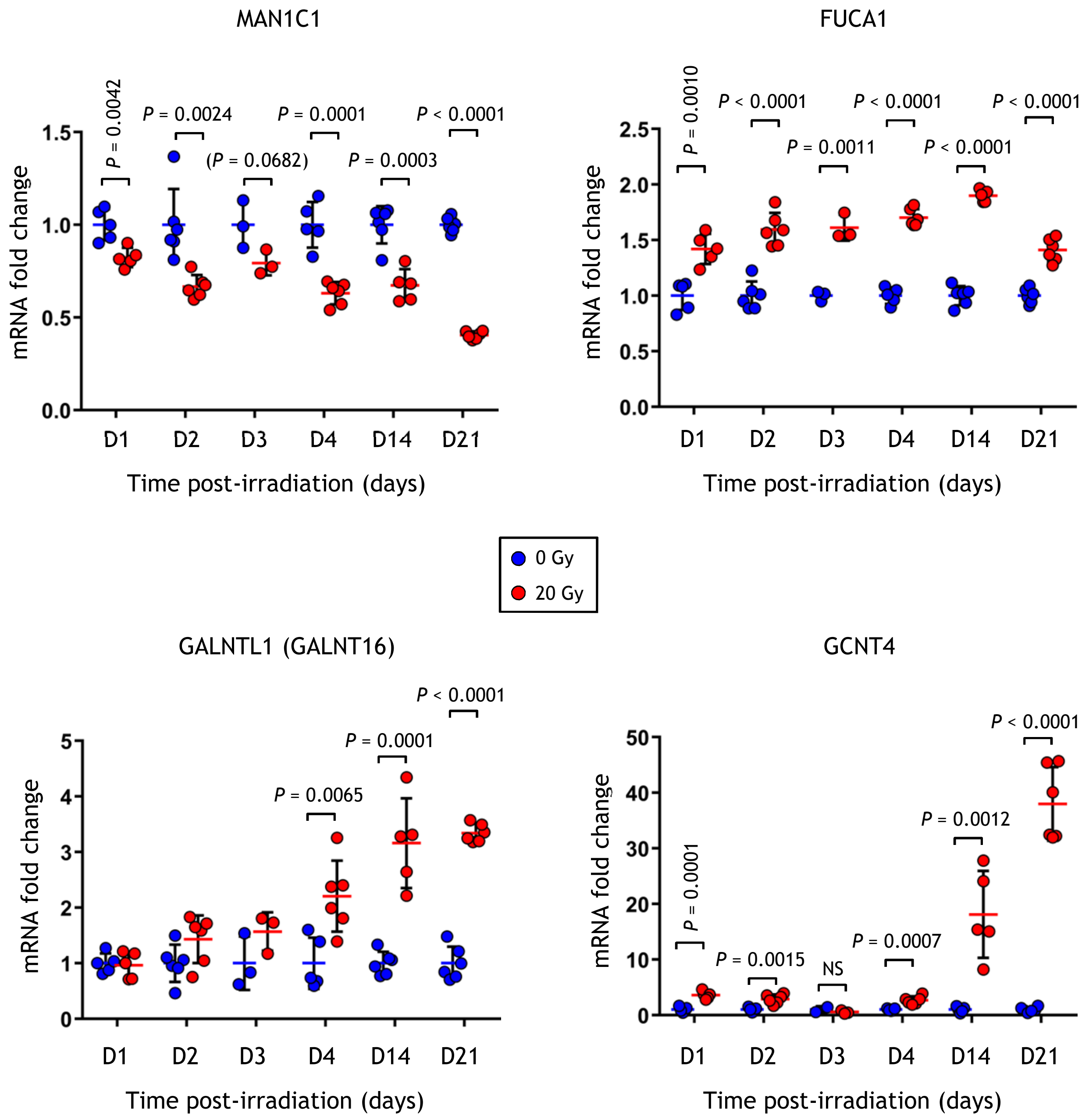

Supplementary Figure S8.Time-course gene expression analysis of MAN1C1, FUCA1, GALNTL1 and GCNT4 identified in the targeted transcriptome analysis (see Fig. 4) following irradiation of HUVECs. Control and 20 Gy-irradiated HUVEC mRNA levels of the 4 genes were measured by real-time quantitative PCR using the SAbiosciences Human Glycosylation RT² Profiler™ PCR Array (1, 2, 3, 4, 14 and 21 days post-exposure) (mean +/- SEM). The experiment was replicated twice (two different cultures) with n=3 repeated measures within the replications for each time point, except for day 3 which was performed once with n=3 repeated measures. Data represent the mean of the two experiments. Data analyzed by two-tailed *t*-test and adjusted *p*-values (Benjamini-Hochberg procedure) (Ctrl vs IR). Ct, ΔCt, $2^{-\Delta Ct}$, fold changes, SD, SEM and *p*-values are displayed in the Supplementary Table S3.
